# Brain Radiotherapy plus Concurrent Temozolomide versus Radiotherapy Alone for Patients with Brain Metastases: A Meta-Analysis

**DOI:** 10.1371/journal.pone.0150419

**Published:** 2016-03-01

**Authors:** Qian Zhao, Qin Qin, Jinglong Sun, Dan Han, Zhongtang Wang, Junjie Teng, Baosheng Li

**Affiliations:** 1 Department of Radiation Oncology, Shandong Cancer Hospital and Institute, School of Medicine and Life Sciences, University of Jinan-Shandong Academy of Medical Sciences, Jinan, Shandong, PR China; 2 Department of Radiation Oncology, Shandong Cancer Hospital and Institute, Jinan, Shandong, PR China; 3 Department of Rehabilitation, Second Affiliated Hospital of Shandong University of Traditional Chinese Medicine, ShanDong, PR China; Taipei Medical University, TAIWAN

## Abstract

**Objective:**

We performed a meta-analysis of randomized clinical trials to compare the efficacy of brain radiotherapy (RT) combined with temozolomide (TMZ) versus RT alone as first-line treatment for brain metastases (BM).

**Methods:**

Medline, Embase, and Pubmed were used to search for relevant randomized controlled trials (RCTs). Two investigators reviewed the abstracts and independently rated the quality of trials and relevant data. The primary outcome was overall survival (OS). Secondary outcomes included progression-free survival (PFS), objective response rate (ORR), and adverse events.

**Results:**

Seven studies were selected from the literature search. RT plus TMZ produced significant improvement in ORR with odds ratio (OR) of 2.27 (95% CI, 1.29 to 4.00; *P* = 0.005) compared with RT alone. OS and PFS were not significantly different between the two arms (OS: HR, 1.00; *P* = 0.959; PFS: HR, 0.73; *P* = 0.232). However, the RT plus TMZ arm was associated with significantly more grade 3 to 4 nausea and thrombocytopenia.

**Conclusion:**

Concomitant RT and TMZ, compared to RT alone, significantly increases ORR in patients with BM, but yields increased toxicity and fails to demonstrate a survival advantage.

## Introduction

Brain metastases (BM) are the most common neurologic complication in cancer patients, occurring in up to 40% of patients affected by solid tumors [1,2]. Brain radiotherapy (RT) including whole-brain radiotherapy (WBRT) and stereotactic radiosurgery (SRS) have been considered standards of care worldwide for BM patients, which lead to a median survival time (MST) of 3 to 6 months [3–5]. The effectiveness of chemotherapy in BM is limited because most the chemical drugs cannot cross the blood-brain barrier [6].

Temozolomide (TMZ) is an orally administered alkylating agent and efficiently crosses the blood-brain barrier [7]. TMZ has demonstrated schedule-dependent activity in the treatment of several solid tumors, including primary brain tumors and metastatic carcinoma [8–9]. Two phase II studies showed that TMZ combination with RT is well tolerated and is associated with significant improvement in quality of life in patients with BM [10,11]. The meta-analysis of Qin et al [12] confirmed that the combination of WBRT plus chemotherapy (including TMZ, carboplatin, and motexafin gadolinium) may increase treatment response rates in patients with BM, therefore TMZ combined with RT can be a reasonable treatment option for patients with BM. However, follow-up studies conclude that the addition of TMZ to RT does not improve survival and has an increased incidence of adverse events compared with RT alone [13,14].

Hence, we conducted the current systematic review and meta-analysis of randomized controlled trials (RCTs) to compare the efficacy of TMZ plus RT to RT alone in patients with BM, with a combined statistical power higher than individual trials.

## Materials and Methods

### Search Methods

To identify potentially eligible trials, we searched electronic data bases (Medline, Embase and Pubmed from 2010 to 2015). For PubMed, the following strategies were used: (1) temozolomide [Supplementary Concept]) AND Brain [Mesh]) AND Radiotherapyn [Mesh], (2) temozolomide AND brain radiotherapy AND [carcinoma OR neoplasms OR neoplasm OR cancer], (3) temozolomide AND [brain OR whole brain] AND [radiotherapy OR irradiation], (4) temozolomide AND [brain radiotherapy or brain irradiation] AND [breast cancer or breast carcinoma], (5) temozolomide AND [brain radiotherapy or brain irradiation] AND [lung cancer or lung carcinoma]. For medline, the following strategies were used: temozolomide AND [brain metastases OR brain metastasis] AND [radiotherapy OR radiation therapy]; The search terms used for Embase were as follows: (1) 'brain metastasis'/exp OR 'brain metastasis' AND ('radiotherapy'/exp OR radiotherapy) AND ('temozolomide'/exp OR temozolomide). (2) [brain metastatic tumour OR brain metastatic tumor OR brain metastasis] AND [radiotherapy OR irradiation OR radiation therapy] AND temozolomide. References of the selected trials, review articles, and relevant books were also checked. Only references published in English were included.

### Selection of Trials

The purpose of the analysis was to compare the efficacy of brain radiotherapy combined with TMZ as the first-line treatment of brain metastasis; therefore, only RCTs comparing RT + TMZ with RT alone as the control arm were used. Studies meeting the following criteria were included in the analysis: (1) the trials must be properly randomized; (2) patients received no previous brain radiotherapy, chemotherapy or surgery; (3) the treatment pattern in each study must be consistent. Brain radiotherapy combined with TMZ was defined as TMZ given the first day and/or within 1 week after radiotherapy. Radiotherapy modalities should be similar in both arms; (4) each study must have reported the outcome data of survival.

Observational studies, retrospective studies and phase I, nonrandomized phase II, phase II trials that were designed mainly to evaluate RT combined with TMZ without significant control arm were excluded. Trials that evaluated RT combined with TMZ versus TMZ were also excluded from the analysis. The global quality score was determined based on the estimate of two reviewers according to the criterion of NSW Department of Health that included the study methods and results, quality of randomization, double-blinding, handling of withdrawals and dropouts; outcome measures, intention to treat analysis and comparability of characteristics at baseline [15]. Trials with global quality scores of B2 (moderate to high risk of bias) or C (high risk of bias) were excluded in the analysis.

### Data Collection and Clinical End Point

Data extraction was conducted by two investigators according to Preferred Reporting Items for Systematic Reviews and Meta-Analyses (PRISMA) guidelines [16, 17]. The following information was collected: age, sex, performance status at the time of random assignment, date of randomization, number of brain metastases, extracranial metastases and information on survival and toxicity. Data were checked for internal consistency and for consistency with published results. Discrepancies between the reviewer’s classifications of publications were resolved by discussion and consensus with the other senior investigator and statistician.

The primary outcome was OS, defined as the time from randomization until death or last follow-up time. Secondary end points were PFS, ORR and adverse events. Both PFS and central nervous system (CNS) progression were included in the analysis. PFS was defined as the time between the dates of randomization to disease progression or death occurred. CNS-progression was measured by contrast-enhanced computed tomography or magnetic resonance imaging (MRI) of the brain according to World Health Organization criteria [18].

### Statistical Analysis

Analysis of efficacy was performed according to the intent-to-treat principle. The odds ratio (OR) was used to quantify the effect of the treatment on tumor response, and its significance was assessed using the Mantel-Haenszel test. The hazard ratio (HR) was estimated to assess the survival advantage of the RT + TMZ as compared with radiotherapy alone. OS and PFS curves were estimated using the Kaplan-Meier methodology and compared using the stratified log-rank test. HR for each group of trials and for all trials together were calculated according to the published methods of Parmar et al. [19] P values were determined by log-rank test. Toxicity variables were dichotomized as severe (grade 3 to 4) and no/mild (grades 0 to 2). The pooled odds ratio with 95% CI was used to compare toxicity rates between arms.

χ^2^ heterogeneity tests were used to test for statistical heterogeneity among trials. I^2^ statistic was used to estimate the percentage of total variation across studies, with an I^2^ value below 50% considered indicative of low heterogeneity [20]. If there was a substantial heterogeneity, a random effect model was tested and the possible clinical and methodological reasons for this were explored qualitatively. All tests were two-sided (*P* = 0.05 was significant). All statistical analyses were performed using STATA version 12.0 software (College Station, TX).

## Results

### Description of the studies

**Fig 1:**
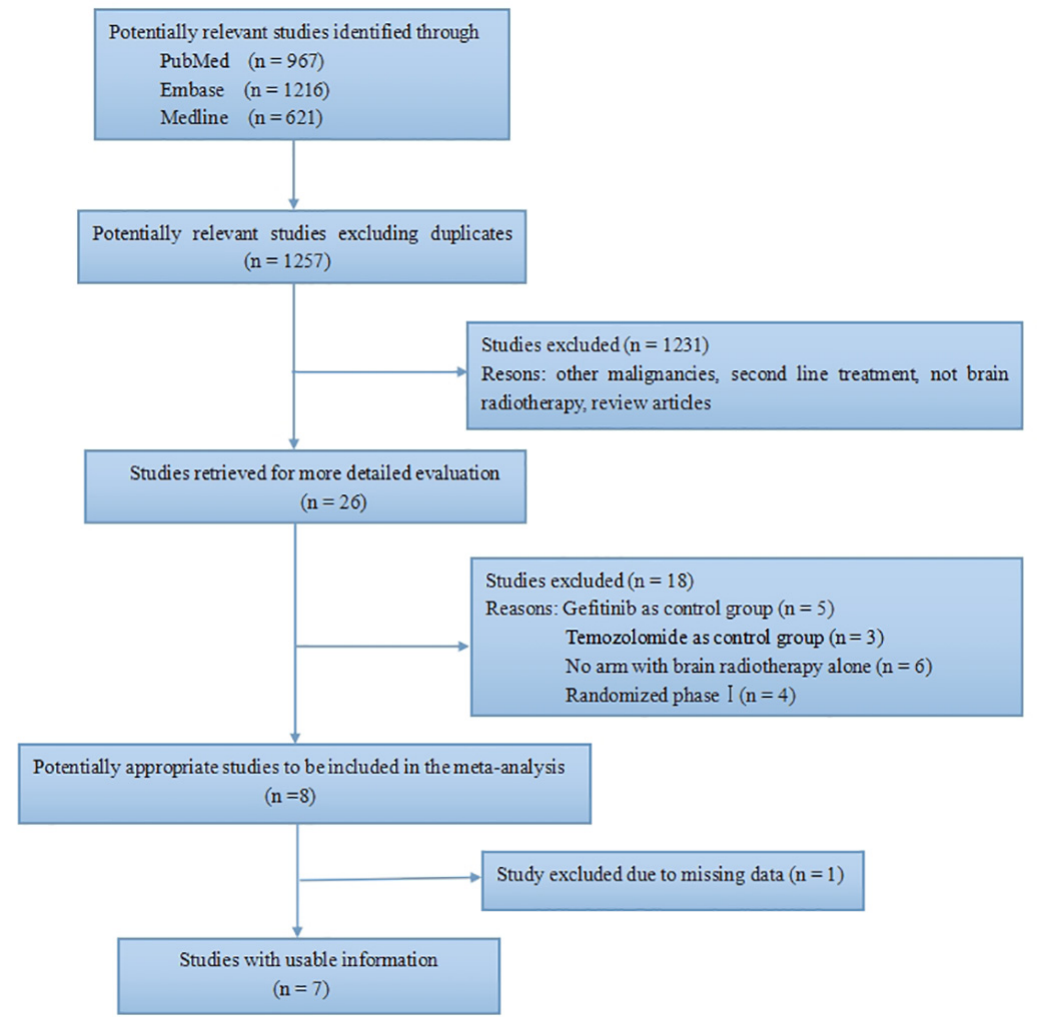
A flow chart showing the progress of trials through the review.

Our search yielded 1257 potentially relevant abstracts. All abstracts were screened, and the full text was retrieved for 26 studies. After reviewing these studies, 18 were eliminated from the analysis for one or more reasons. The selection process and reasons for exclusion are detailed in Fig 1. Six trials were eliminated because no RT control was employed. Eight trials were excluded because the control arm was TKI or TMZ. Thus, the results of this meta-analysis are based on seven trials, including six phase II trials, and one phase III trial. The characteristics of the trials selected for this meta-analysis are presented in Table 1 [21–27].

**Table 1:** Trial Characteristics.

Study	Year	Phase	No.of Patients	Histology	Trial * quality	Primary end point	BRT + TMZ		BRT alone
							TMZ	BRT	
Antonado et al^21^	2002	II	48	BCLC	B1	Radiologic response	During WBRT 75 mg/m2/d After WBRT 200 mg/m2/d 5 days every 28 days to fasting patients for a maximum of six additional cycles.	40Gy/20f	40Gy/20f
Verver et al^22^	2005	II	82	BCLC	B1	PFS	During WBRT 75 mg/m2/d After WBRT 200 mg/m2/d for 5 days (150 mg/m2 in heavily pretreated patients) every 28 days.	30Gy/10f	30Gy/10f
Chua et al^23^	2010	II	95	NSCLC	B1	Overall survival	During WBRT 75 mg/m2/d After WBRT 75 mg/m2/d 5 days every 28 days	30Gy/10f	30Gy/10f
Carlos et al^24^	2012	II	55	BCLC	B1	ORR	TMZ was administered 1 h before each WBRT, at a fixed dose of 200 mg on Mondays, Wednesdays and Fridays and at a fixeddose of 300 mg on Tuesdays and Thursdays.	30Gy/10f	30Gy/10f
Hasser et al^25^	2013	II	35	NSCLC	B1	Safety and toxicity	During WBRT 75 mg/m2/d Two weeks after WBRT, TMZ 100 mg/m2 every 28 days until unacceptable toxicity or progression of disease for up to six cycles	40Gy/20f or 30Gy/10f	40Gy/20f or 30Gy/10f
Sperduto et al^26^	2013	III	84	NSCLC	B1	Overall survival	During WBRT 75 mg/m2/d After completion of WBRT and SRS, 150 mg/m2/d for 5 days/month for as long as 6 months	37.5 Gy/15f	37.5 Gy/15f
Cao et al^27^	2014	II	100	BC	B1	ORR	During WBRT 75 mg/m2/d After WBRT 75 mg/m2/d 5 days every 28 days	30Gy/10f	30Gy/10f

Gy, Gray; fr, fraction; BC, breast cancer; LC, lung cancer; NSCLC, non-small lung cancer; BRT, brain radiation therapy; TMZ, temozolomide; WBRT, whole brain radiation therapy; PFS, progression-free survival; ORR, objective response rate.

*Global score: A, low risk of bias; B1, low-moderate risk of bias; B2, moderate-high risk of bias; C, high risk of bias.

A total of 499 patients are included in the meta-analysis, with 257 patients in the RT group and 242 patients in the RT plus TMZ group. Patient characteristics are summarized in Table 2. Patients who were included followed the eligibility criteria defined by each trial. The mean age of the combination treatment group and the RT group were 59.8 and 59.2 years, respectively. In all trials except for the Radiation Therapy Oncology Group (RTOG) 0320 [26], the recommended dose of cranial irradiation ranged from 30 to 40 Gray (Gy) given in 10 to 20 fractions, which corresponds to a dose of 2 to 3 Gy per fraction [21–25,27]. RTOG 0320 used a dose of 37.5 Gy delivered for 15 fractions with the addition of SRS to WBRT.

**Table 2:** Patient Characteristics.

Characteristic	BRT + TMZ (n = 257)		BRT alone (n = 242)	
	No.	%	No.	%
Sex				
Male	130	51	128	53
Female	127	49	114	47
Median age, years	59.8	-	59.2	-
Range	37–79	-	29–81	-
KPS				
≤	162	63	162	68
≥	51	20	40	16
Data not supplied	44	17	40	16
Primary tumor site				
Breast cancer	79	31	73	30
NSCLC and others	176	68	168	69
Unknown	2	1	1	1
Extracranial metastases				
Yes	147	57	144	60
No	88	34	85	35
Data not supplied	22	9	13	5
RPA class				
I	35	14	31	13
II	105	41	95	39
III	20	8	18	8
Data not supplied	97	37	98	40

No., number; BRT, brain radiation therapy; TMZ, temozolomide; KPS, Karnofsky performance status; NSCLC, non-small cell lung cancer; RPA, recursive partitioning analysis.

There was a statement regarding both randomization and withdrawals in reports on all seven trials.

### Short-term outcome

**Fig 2:**
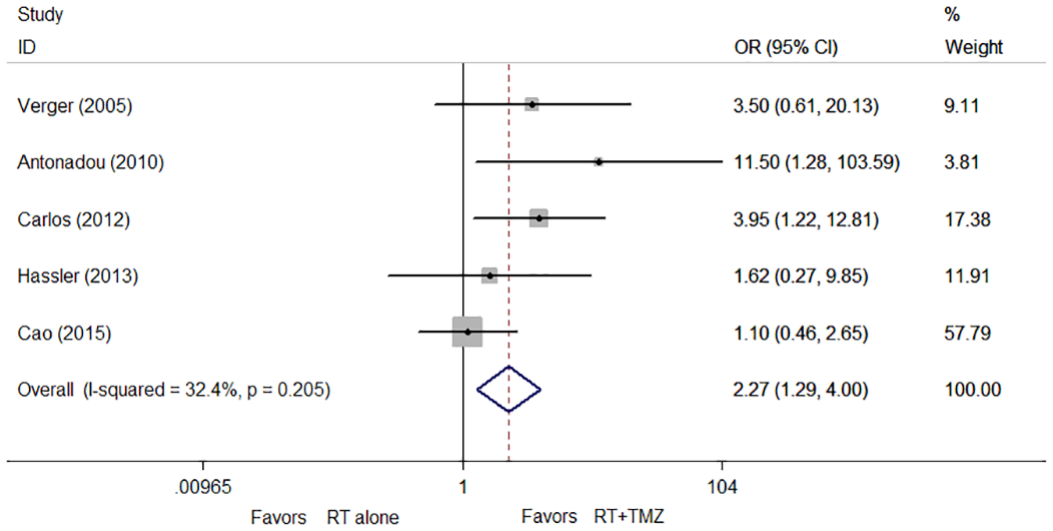
Hazard ratios (HR) and 95% confidence intervals (CIs) for objective response rate (ORR).

Objective response rates (ORR) were significantly greater for concomitant RT plus TMZ compared to RT alone (OR, 2.27; 95% CI, 1.29 to 4.00; *P* = 0.005; Fig 2). There was no statistically significant heterogeneity among the five trials (*P* = 0.205; I^2^ = 32.4%).

### Survival

**Fig 3:**
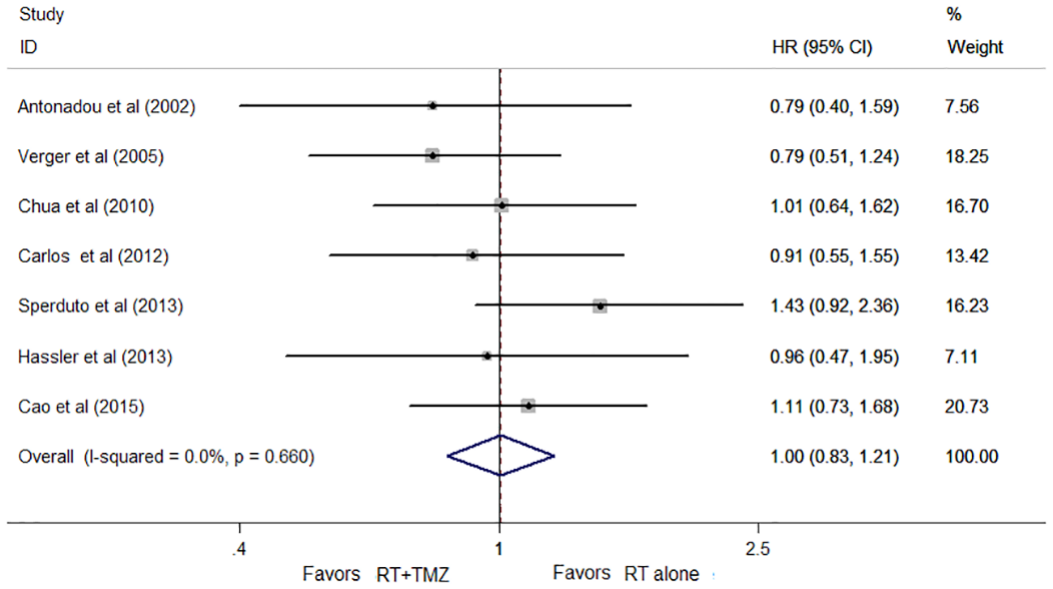
Forest plot of overall survival (OS) for patients with brain metastases (BM).

The survival analysis of the seven trials had a median follow-up time of 5.6 months. No difference was observed between the two arms in terms of OS (HR, 1.00; 95% CI, 0.83 to 1.21; *P* = 0.959; Fig 3). Concomitant RT and TMZ yielded a 12% increase in 1-year survival compared to RT alone. This difference was not statistically significant (HR, 0.88; 95% CI, 0.66 to 1.16; *P* = 0.359). There was no statistically significant heterogeneity in the HRs for OS and 1-year survival from the individual trials (OS: *p* = 0.660; I^2^ = 0%, 1-year OS: *P* = 0.677; I^2^ = 0%).

**Fig 4:**
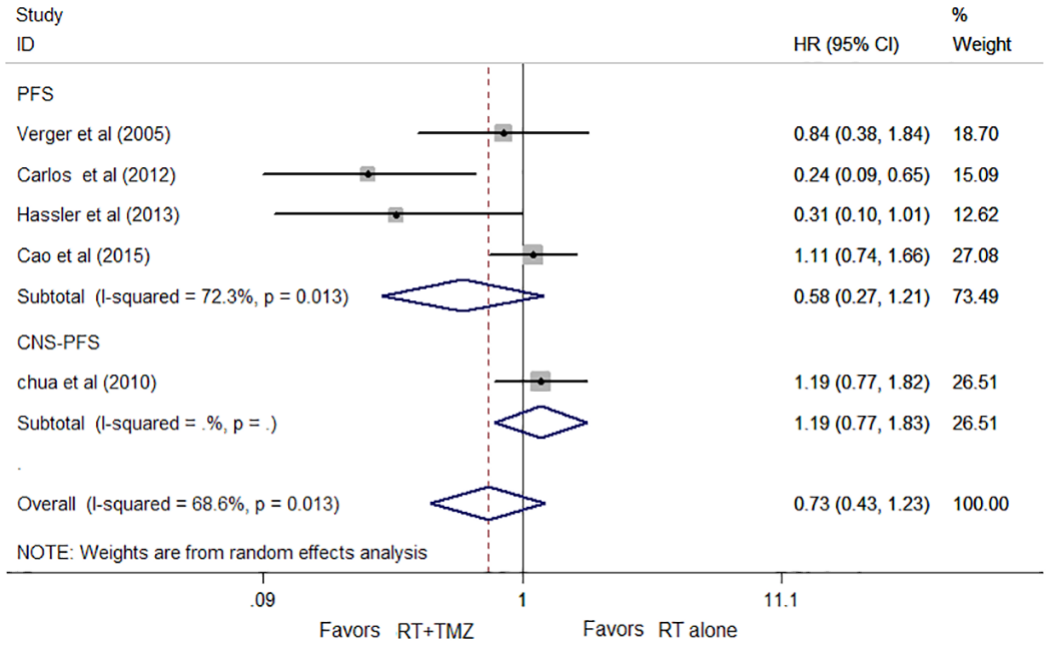
Hazard ratios (HR) and 95% CIs for progression-free survival (PFS).

The progression-free survival (PFS) data were available for five trials and included 367 patients. Concomitant RT and TMZ treatment was associated with a 27% improvement in PFS compared to RT alone, but this difference was not statistically significant (HR, 0.73; 95% CI, 0.43 to 1.23; *P* = 0.232; Fig 4). There was some evidence of heterogeneity between the trials (*P* = 0.013; I^2^ = 68.6%). The analysis of three trials (the study of Carlos et al [24] excluded) resulted in considerably lower heterogeneity with an I^2^ value of 39.6% (χ^2^ test for heterogeneity *P* = 0.174). Exclusion of the data of Carlos et al [24] did not alter our results or conclusions (HR, 0.96; 95% CI, 0.658 to 1.41; *P* = 0.846). Funnel plot and rank correlation test regarding survival confirmed the absence of publication bias (Z = 0.69; *P* = 0.53).

### Patient Subgroups

**Fig 5:**
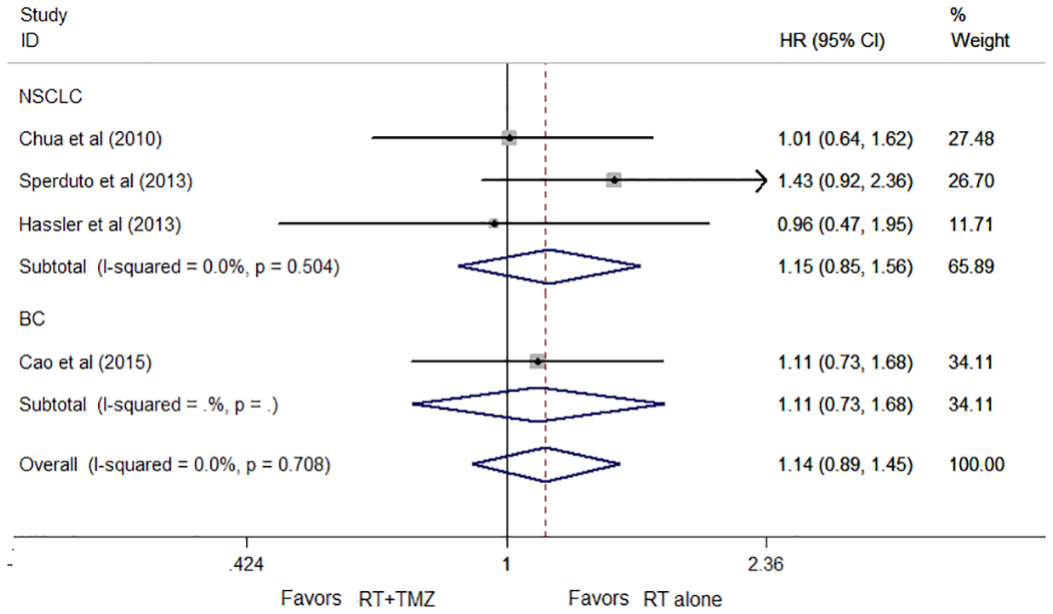
Forest plot of subgroup analysis by cancer type for OS.

There were no statistically significant differences in the effects on overall survival between subgroups defined by the primary tumor site (non-small lung cancer (NSCLC) *P* = 0.354; breast cancer (BC) *P* = 0.624). Hence, the difference in overall survival was also nonsignificant (HR, 1.14; 95% CI, 0.89 to 1.45; *P* = 0.299; Fig 5). No apparent statistical heterogeneity was observed between the two subgroups with an I^2^ value of 0%. (χ^2^ test for heterogeneity *P* = 0.886).

### Toxicity

**Fig 6:**
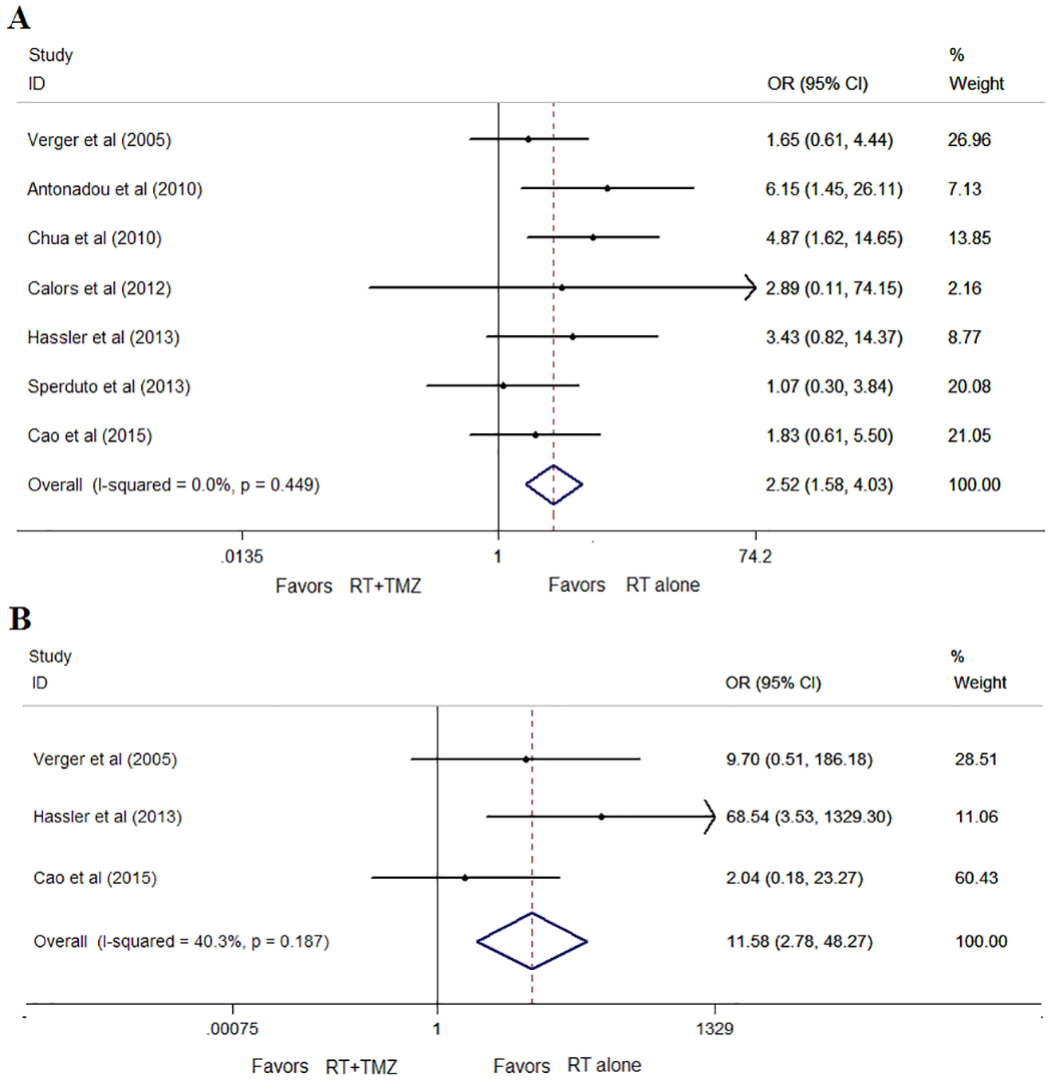
Forest plot of severe toxicities: (A) nausea; (B) thrombocytopenia. *Favors RT+TMZ = Lower risk in RT+TMZ.

As expected, patients treated with RT plus TMZ had significantly more grade 3 to 4 nausea (25% v 41%; OR, 2.52; 95% CI, 1.58 to 4.03; *P*<0.001, I^2^ = 0%; Fig 6A). The concomitant group also had significantly increased grade 3 to 4 thrombocytopenia compared to the RT group (OR, 11.58; 95% CI, 2.78 to 48.27; *P* = 0.001, I^2^ = 40.3%; Fig 6B).

No significant difference in the number of patients with grade 3 to 4 fatigue was observed between the two modalities (OR, 1.42, 95% CI, 0.73 to 2.77; *P* = 0.30). With regard to headache, insomnia, and balance disorders, no difference was observed with ORs of 1.46 (95% CI, 0.73 to 2.91; *P* = 0.28), 1.04 (95% CI, 0.36 to 3.03; *P* = 0.94), and 1.98 (95% CI, 0.82 to 4.78; *P* = 0.13), respectively. Brain necrosis and neurologic death were not reported in any of the included trials.

## Discussion

The current meta-analysis showed that the addition of TMZ to RT was associated with a significant improvement in ORR. This increase in ORR did not translate into an increase in OS and PFS compared with RT alone, but there was a statistically significant increase in toxicity. Based on these results, concomitant treatment is not recommended.

Several nonrandomized studies that we could not include were in favor of TMZ plus RT with median overall survival of 12 months and a high level of satisfaction for the quality of life [12–13,28–29]. However, individually, only one out of seven RCTs included in this meta-analysis demonstrated a slight improvement in survival following the addition of TMZ to RT, with median survival times of 8.6 and 7 months in the TMZ plus RT and RT arm, respectively [21]. In four RCTs that compare RT to concomitant TMZ and RT, the OS was lower in patients that were treated with combination TMZ and RT [23,25–27]. Similarly, our meta-analysis confirmed these findings and suggested that there was no significant improvement in survival between patients treated with RT plus TMZ, or RT without TMZ, based on a direct comparison.

The meta-analysis of Bai et al [30] examined ORR and mOS of WBRT and TMZ in treatment for BM patients. The significant improvement of ORR in WBRT+TMZ arm was consistent with our study. Yet different results of survival were showed. Their study demonstrated a significantly improved survival in WBRT plus TMZ arm, which was inconsistent with our findings. It is important to remember mean difference of mean overall survival (mOS) between two groups was established to analyze the trend of OS. However, the overall HR was used to estimate the improvement of OS in our analysis. Due to the differences of the statistical methods of survival, the results of OS were different.

It is important to note in our study that a significant benefit of ORR was found in the concomitant group compared to the RT group. Several trials have demonstrated that TMZ plus WBRT yielded more favorable results reaching ORRs from 53% to up to 96% in patients with BM [23–27]. Despite these observed benefits, the improvement of survival in the enrolled trials tends to be similar and insignificant. The apparent discrepancies between results of ORR and OS can be explained by considering several factors. First, other therapies at any stage, such as anti-HER-2 therapies of breast cancer and targeted therapies of lung cancer, may affect the long-term efficacy and sensitivity of treatment [31–33]. The overall survival benefit of radiotherapy combined with TMZ is diluted and may be negligible when the patients are treated with other therapies. However, the implication here does not affect the patterns of response for patients with BM. Second, prognostic factors, such as the primary tumor site, recursive partitioning analysis (RPA) class, and number of metastases, are important factors influencing survival in patients with BM [34–38]. These factors have not been considered to play a role in the response rate, but imply considerable differences in OS and PFS. Third, the objective response is an endpoint in trials that assess relative short-term efficacy of treatment. The differences in survival and objective response are partly caused by the relative efficacy of radiotherapy with concurrent TMZ and the severe pattern of treatment-related toxicities [38–40]. The survival benefits involved a small part of patients who responded to the treatment and tolerated the increase in toxicity [40–41]. Thus, the increase in ORR in radiotherapy combined with TMZ did not translate into an increase in OS and PFS compared with RT alone.

Toxicity is particularly relevant in the treatment of brain metastases, given the potential negative impact on benefit and quality of life. We found that there was significantly increased thrombocytopenia and nausea of grade 3 or higher in the concomitant group compared to the RT group. These toxicities related to the treatment may significantly impair quality of life, but subside after treatment. With respect to neurocognitive outcomes, only the Hassler study evaluated the impact of WBRT and TMZ on cognition, and suggested that grade 3 or higher cognitive dysfunction were more common in the combination group [25]. The present meta- analysis showed that there were no statistically significant differences in other neurologic symptoms, such as balance disorders and insomnia, in the combination and RT groups. It is unknown how this difference would change with a larger number of comparisons.

There are several potential limitations to our analysis, notably, the different treatment schedules among the included trials. In six of the seven included trials, patients assigned to the concomitant group were required to continue TMZ at different doses and for different numbers of cycles after the radiotherapy. Despite this, the analysis showed remarkably similar results of OS and PFS between the enrolled trials. This study provided the comparison of RT and RT+TMZ, and suggests that the difference in approaches may be negligible. The other limitation is mostly related to the heterogeneity of PFS among the enrolled trials. Despite the heterogeneity between the study by Carlos et al [24] and the other trials, exclusion of these data did not alter our results or conclusions. The differences in number of metastases might be potential cause of this heterogeneity of PFS in patients with BM, but we could not test this hypothesis in our meta-analysis. Furthermore, a widely acknowledged difficulty in RCTs was control of the confounding factors. Because of factors such as the primary tumor site, recursive RPA class, and treatment intensification, the data were not consistent across the trials and in the integrated analysis, resulting in limited clinical heterogeneity. The ability of this study to analyze these factors was limited because of low statistical power and the limitation of published data. Further analysis should be performed to confirm the factors observed here.

In conclusion, the addition of TMZ to RT, compared to RT alone, improved the ORR of patients with BM, without any beneficial effect on OS and PFS, at the cost of manageable increased thrombocytopenia and gastrointestinal tract reaction. Randomized evidence available for our meta-analysis does not support the use of concomitant RT and TMZ as part of the standard treatment of patients with BM.

## Supporting Information

S1 File Original data. (ZIP)

S1 PRISMA Checklist PRISMA checklist. (DOC)
